# Optimization of the green synthesis of gold nanorods using aqueous extract of peeled sour guava as a source of antioxidants

**DOI:** 10.1371/journal.pone.0313485

**Published:** 2025-01-08

**Authors:** M. Camila Patiño-González, Claudia E. Echeverri-Cuartas, Sandra Torijano-Gutiérrez, Sandra Milena Naranjo-Rios, Natalia A. Agudelo

**Affiliations:** 1 Grupo de Investigación de Ingeniería Biomédica-GIBEC, Escuela de Ciencias de la Vida y Medicina, Programa de Ingeniería Biomédica, Universidad EIA, Colombia; 2 Grupo de Investigación en Síntesis Orgánica, de Polímeros y Biotecnología Aplicada-SINBIOTEC, Escuela de Ingeniería y Ciencias Básicas, Universidad EIA, Colombia; Universidad Tecnica de Ambato, ECUADOR

## Abstract

Obtaining gold nanorods (AuNRs) through biosynthesis is an alternative that replaces the traditional use of ascorbic acid with chemical compounds such as polyphenols, owing to their notable antioxidant properties. Therefore, we developed an AuNR biosynthesis method using an aqueous extract of sour guava (*Psidium araca*). Initially, a study was conducted to determine the antioxidant capacity of different parts of the fruit (pulp and peel) over 14 days. Four colorimetric techniques were used: total phenol, ABTS (2,2-azino-bis-3-ethylbenzothiazoline-6-sulphonic acid), FRAP (ferric reducing antioxidant power (FRAP), and DPPH (1,1-diphenyl-2-picrylhydrazyl). Subsequently, in stage 2, the selected aqueous extract was used, and two response surface designs were performed. The objective of this study was to find a model equation that would indicate the optimal parameters for obtaining AuNRs with a surface plasmon band at 808 nm, with possible applications in the health field. The results of the antioxidant capacity experiments were analyzed in Minitab^®^ using a multilevel factorial design, and the peel exhibited the highest antioxidant capacity. Subsequently, the biosynthesis of AuNRs proceeded using a 5-factor response surface experimental design as input variables (concentration in mM of gold, silver, extract, NaBH_4_, and reaction time in hours) and longitudinal plasmon (LSPR) as output variables. The AuNRs were approximately 30 nm in size with an LSPR between 700 and 800 nm. Statistical model evaluation revealed a dependence between gold and time and gold–silver factors. Finally, antioxidant capacity was used to select the part (peel or pulp) of sour guava that could be used as a weak reducing agent. Moreover, the utility of surface-response methodology was explored to optimize the synthesis of AuNRs using green agents.

## 1. Introduction

Gold nanorods (AuNRs) are nanometric materials (1–100 nm) with anisotropic properties, which indicate that, owing to their geometry, their optical, morphological, and chemical properties constantly change with the direction and parameters of the synthesis [1, 2]. These nanoparticles presented two surface plasmon bands (SPR) along the UV–Vis spectrum, which were associated with the interaction between the confined free electrons located on the surface of the cross-section (TSPR) and longitudinal (LSPR) of the nanorod [1]. The transverse band generally manifests as a maximum absorbance at 520 nm [1, 3]. By contrast, the longitudinal section band is characterized by maximum absorbance in the near-infrared (NIR) range of 600–1200 nm [1, 4]. Therefore, AuNRs can be used in medical applications such as photothermal therapy [2, 5] functionalization for delivery systems, and diagnostic imaging [5].

Nanoparticles can be obtained using two methods: with and without seeds [4, 6]. The first consists of chemical synthesis, which initially forms Au (0) nanoparticles or “seeds” of 1–5 nm from the reduction of chloroauric acid (HAuCl_4_•3H_2_O) and sodium borohydride (NaBH_4_) in a hexadecyltrimethylammonium bromide (CTAB) micellar medium. In the second sample, a growth solution was prepared to which different mediators were added to form nanorods, followed by the seeds synthesized in the first step. The components of this second solution were mainly silver nitrate (AgNO_3_) and hydrochloric acid (HCl) as growth controllers, and ascorbic acid (AA) as a weak reducer of the solution [4, 7, 8].

Subsequently, a seedless method was proposed consisting of the formation of AuNRs in a single step using the above reagents [9]. The difference between these two methods lies mainly in that the seedless method is a more controlled process than the other method since the latter depends on several factors that affect the growth of the nanostructure, for example, the aging of the seeds [9, 10]. By contrast, the seedless method has been used in the biosynthesis of AuNRs, which is defined as an alternative for synthesizing AuNRs through chemical molecules derived from fruit and vegetable extracts, known as polyphenols [11]. In the biosynthesis of AuNRs, the use of pure polyphenols such as resveratrol [12], catechins [13], and gallic acid [14] has been reported, which replace AA in traditional synthesis owing to their antioxidant properties that generate a weak reduction effect on synthesis [11]. However, the use of aqueous extracts, that is, extracts prepared only with water and fruits or vegetables without any extraction of active compounds using an organic solvent, in the biosynthesis of AuNRs has not yet been studied because still no relationship has been revealed between the antioxidant properties of the extract and the effect of reduction and production of AuNRs. Furthermore, no established method indicates the concentration relationship between the reagents and the extract.

Considering that antioxidant properties are a determining factor for the selection of an aqueous extract, it has been reported that sour guava (*Psidium araca*), a tropical fruit characteristic of Colombia, has a high antioxidant capacity owing to the presence of different types of polyphenols, such as phenolic acids and flavonoids [15]. Research on the biosynthesis of AuNPs using plant extracts has shown that these extracts contain many active phytochemical compounds, such as polyphenols (phenolic acids, flavonoids, lignans, and tannins), reducing sugars, polysaccharides, glycosides, alkaloids, triterpenoid saponins, proteins, steroids, triterpenoids, saponins, fatty acids, and organic acids [16]. However, only three types of active phytochemical compounds reduce Au ions to obtain and stabilize NPs: polyphenols, reducing sugars, and proteins [16, 17].

The synthesis of gold nanostructures using sour guava has not yet been reported. This fruit has not been used to synthesize other types of metallic nanostructures such as silver, copper, or iron particles. However, from the same family as guava but not specifically sour guava, *Psidium guajava* (guava) produces metallic nanostructures. For example, silver nanoparticles (AgNPs) have been prepared via a green synthesis method using guava fruit extract as a reducing agent and stabilizer [18]. In addition, stable polyshaped AuNPs were obtained using aqueous extracellular anti-malignant guava (*Psidium guajava*) [19]. Other particles, such as zero-valent iron nanoparticles, have been synthesized from *Psidium guajava* in the range of 2–5 nm [20]. Kumar *et al*. biosynthesized tin oxide nanoparticles using *Psidium Guajava* leaf extract for photocatalytic dye degradation [21]. Recently, the green synthesis of ZnO oxide nanoparticles with antibacterial applications using *Psidium guajava* leaf extract has been reported [22].

In summary, according to previously reported research, the nanostructures studied were obtained from the leaf extracts of *Psidium guajava* but not the fruit. Therefore, in this study, sour guava fruit was chosen as the aqueous extract of interest to obtain AuNRs with potential health applications. To achieve this, the antioxidant properties of sour guava were evaluated separately (pulp and peel), and the extract with the highest antioxidant capacity was selected as a weak reducing agent using colorimetric techniques, such as total phenols, ABTS (2,2-azino-bis-3-ethylbenzothiazoline-6-sulphonic acid), FRAP (ferric reducing antioxidant power (FRAP), and DPPH (1,1-diphenyl-2-picrylhydrazyl).

Subsequently, in developing a protocol to obtain AuNR by biosynthesis with sour guava extract, the Minitab^®^ software was used to propose a response surface experimental design, using the concentration of the reagents and the reaction time as factors of the input variables (HAuCl_4_•3H_2_O, NaBH_4_, AgNO_3_, extract, and time). The response or output variable was the wavelength obtained from the longitudinal plasmon band (LSPR) of the AuNRs. The resulting model was analyzed with a 95% confidence interval, significant differences between factors were identified, and the optimal response of the model was determined by estimating a longitudinal band around 808 nm.

## 2. Materials and methods

### 2.1 Materials

*Psidium araca* was obtained from Montelíbano, Colombia. Table 1 presents the reagents used to synthesize the nanorods without any additional purification steps. The solutions were prepared using H_2_O_d_.

**Table 1:** Reference of the chemical for the AuNR biosynthesis.

Chemical	Manufacturer	Reference
HAuCl_4_•3H_2_O	Sigma-Aldrich	520918
CTAB	Sigma-Aldrich	H9151
AgNO_3_	Sigma-Aldrich	209139
HCl	Merck	100317
NaBH_4_	Sigma-Aldrich	452882

Folin-Ciocalteu reagent (Sigma-Aldrich), gallic acid (Sigma-Aldrich), and Na_2_CO_3_ (Merck) were used for the total phenolic assays. Antioxidant activity assays were performed using the ABTS assay kit (Abexxa), FRAP MAK369-1KT kit (Sigma-Aldrich), 1,1-diphenyl-2- picrylhydrazyl (DPPH), and methanol (PanReac AppliChem).

No special permissions were required for this study. All methods were developed at the EIA University.

### 2.2 Obtention of *Psidium araca* extract

The fruit (500 g) was washed with distilled water to remove dust and soil particles. The pulp and peel were then separated, and 50 g of each part was added to 200 mL of distilled water. The samples were homogenized in a blender for 10 min. Subsequently, the samples were served in a thermal bath between 50 and 60 °C for 20 min at 400 rpm, allowed to rest at room temperature for 2 h, and then stored at 4 °C for 24 h. Finally, the samples were centrifugated for 10 min at 9000 rpm and 25 °C (SL 8/8R centrifuge, Thermo Fisher Scientific), and the resulting solid was discarded, preserving the supernatants labeled as aqueous extract. Samples were stored at -20 °C and lyophilized (VirTis SP Scientific) at -50 °C and 150 mTorr. Finally, the solid extracts of the pulp and peel of *Psidium araca* were obtained.

### 2.3 Total phenols and antioxidant capacity of *Psidium araca* extract over time

The variation in antioxidant capacity over time was determined for the solid extracts of the peel and pulp over 14 days (days 0, 7, and 14). Colorimetric technique assays for total phenols, ABTS, FRAP, and DPPH were used.

- ***Total phenols*:** Total phenols in the aqueous extracts were determined using the Folin-Ciocalteu method reported in previous studies [15, 23]. A calibration curve was obtained using gallic acid as the standard reagent in the concentration range of 40–200 mg/mL. The samples were then prepared at 120 mg/mL in H_2_O_d_ to obtain the total phenol content in the extracts. Subsequently, a solution was prepared in a conical tube with 7.5 mL of H_2_O_d_, 500 μL of Folin-Ciocalteu 2 N, and 500 μL of the sample. Then, 1.5 mL of sodium carbonate Na_2_CO_3_ at 20% m/v was added and mixed in a vortex shaker for 1 min. Subsequently, the tubes were stored in an oven for 2 h at 37 °C. Finally, 250 μL of the samples were served in triplicate in a 96-well microplate, and the absorbance of each well was measured at 737 nm in a Multiskan GO spectrophotometer (Thermo Scientific). Finally, using Eq 1, the total phenol content was determined based on the mass of gallic acid in 100 g of fresh fruit.
$$Total\;phenols=\frac{mg\;gallic\;acid}{100\;g\;fresh\;fruit}$$

**Equation 1.** Amount of total phenols

- ***2*,*2-Azino-bis-3-ethylbenzothiazoline-6-sulphonic acid assay (ABTS)*:** This colorimetric technique was used to calculate the antioxidant activity of the extracts through the reaction of the ABTS^+^ cation in the presence of antioxidant agents [24]. In the current study, the pulp and peel extracts of *Psidium araca* were evaluated. A calibration curve was obtained using trolox as the test standard solution in the concentration range of 0.4–2 mmol/L. To determine the antioxidant activities of the extracts, samples were prepared at a concentration of 120 mg/mL using H_2_O_d_. Then, in a 96-well microplate, 10 μL of the sample was added in triplicate. Subsequently, 200 μL of the kit buffer was added, and the initial absorbances of the samples were read at 660 nm (absorbance 1). Then, 20 μL of the chromogenic agent was added to each of the wells, and the microplate was stored in an oven for 5 min at 37 °C, protecting it from light. Finally, the absorbance of the samples was measured at 660 nm (absorbance = 2). This result was related to the Trolox mass equivalent to 100 g of fruit (Eq 2).
$$ABTS=\frac{\mu mol\;Trolox\;equiv}{100\;g\;fesh\;fruit}$$

**Equation 2.** Antioxidant activity by ABTS

The μ*mol Trolox equiv* was determined by Eq 3.


$$\mu mol\;de\;Trolox\;equiv=\left(\Delta AABlank–\Delta AAsample-b\right)\;a\times ff\times 1000\times V$$

**Equation 3**. Calculation of the amount of Trolox equivalent

where:

*ΔAA sample*: *absorbance 2*—*absorbance1*

*ΔAA blank*: *the absorbance of the blank at 660 nm before and after adding the chromogenic agent*

*a*: *calibration curve slope*

*b*: *calibration curve intercept*

*ff*: *assay dilution factor*

*V*: *solution volume (L)*

- ***Ferric Reducing Antioxidant Power (FRAP)*:** This method was used to determine the antioxidant activity of *Psidium araca* extracts using the reducing power of ferric ions [24]. The calibration curve was determined using Fe^3+^ solution standard 2 mM as the standard reagent. A curve was constructed using concentrations ranging between 4 and 10 nmol/well. Furthermore, to determine the antioxidant activity of the extracts, aqueous samples were prepared at a concentration of 120 mg/mL in H_2_O_d_. Furthermore, 380 μL of FeCl_3_, 3.04 mL of buffer, and 380 μL of FRAP PROBE were mixed at 300 rpm for 5 min, and this solution was called “reaction mixture.” Then, in a 96-well microplate, 10 μL of each sample in triplicate and 190 μL of the reaction mixture were added to each well and redispersed. Subsequently, the microplate was stored in an oven at 37 °C for 1 h and protected from light. The absorbance of each well was then measured at 594 nm. This result was related to the amount of reduced nmol Fe^2+^(nanomole of iron II) by a gram of fresh fruit (Eq 4).
$$FRAP=\frac{nmol\;Fe^{2+}}{g\;fresh\;fruit}$$

**Equation 4.** Antioxidant activity by FRAP

The nmol Fe^2+^ was determined by Eq 5

$$nmol\;Fe^{2+}=B\times D\times 10^{6}$$

**Equation 5.** Calculation of the amount of nmol Fe^2+^

where B is the amount of ferrous ammonium sulfate calculated using the equation of the straight line of the calibration curve and the absorbance of each extract at 594 nm, and D is the dilution factor.

- ***1*,*1-Diphenyl-2-Picrylhydrazyl assay (DPPH)*:** This technique is based on the scavenging of free radicals present in antioxidant agents in the presence of DPPH [25]. To carry out the assay, 0.9858 mg of DPPH was diluted in a 25 mL volumetric flask. The volume was made up of methanol, and the absorbance was measured at 515 nm. By contrast, aqueous extracts were prepared at concentrations of 0.05, 0.1, 0.15, 0.20, 0.25, and 0.30 mg/mL in H_2_O_d_. Then, 700 μL of each sample and 700 μL of the DPPH solution were added to a dark container. The mixture was vortexed and allowed to stand for 20 min. The absorbance of the samples was measured at 515 nm. Finally, the curve equation was obtained, and the inhibition percentage was calculated (Eq 6) to determine the efficiency coefficient (EC50), which indicates the concentration of the radical when it is reduced to at least 50% [24, 25].
$$\%Inhibition=\frac{Abs_{control}-Abs_{sample}}{Abs_{control}}\times 100$$

**Equation 6.** Percentage of inhibition determined by reduction of the DPPH radical

A complete factorial design was used to validate the results of the different antioxidant activity assays in the Minitab^®^ software, considering two factors: time (days 0, 7, and 14) and section of the fruit (peel and pulp). Significant differences were observed between the factors on each evaluation day. Finally, the extract with significant antioxidant capacity was selected.

### 2.4 Synthesis and characterization of AuNR by the seedless method using *Psidium araca* extract

For the biosynthesis of AuNRs, the seedless method was used, and the reaction time and concentrations of the reactants HAuCl_4_•3H_2_O, AgNO_3_, NaBH_4_, and the aqueous extract were defined according to the response surface experiment design, which will be described later.

Ten milliliters of 50 mM CTAB was mixed with 100 μL of HAuCl_4_•3H_2_O under a thermal bath at 50 °C. Then, 100 μL of AgNO_3_ and 60 μL of HCl were added. Afterward, 800 μL of the aqueous extract was added and left stirring at 400 rpm for 1 h. Finally, 25 μL of NaBH_4_ was added, and the solution was stored in an oven at 50 °C for the reaction time defined in the experimental design. The particles were purified by centrifuging 10 mL of the dispersion for 3 h at 7,000 rpm. The supernatant was discarded and the pellet was redispersed in 5 mL of distilled water.

- **Experiment design**

The time and interaction between the concentration of HAuCl_4_•3H_2_O, AgNO_3_, NaBH_4_, and *Psidium araca* extract were optimized through a composite central response surface experimental design. The input and output factors for each variable and the minimum and maximum levels are listed in Table 2.

**Table 2:** Input (I) and output (O) variables for the response surface experimental design.

Variable	Type	Central levels		Axial levels	
**HAuCl**_**4**_ **•3H**_**2**_ **O (mM)**	I	40	60	33	67
**NaBH**_**4**_ **(mM)**	I	1	10	0.55	12.45
**AgNO**_**3**_ **(mM)**	I	10	30	3	37
**Extract (mg/mL)**	I	10	30	10	37
**Time (h)**	I	24	48	15.6	56.4
**LSPR (nm)**	O	680	800	***x***	***y***

Subsequently, the design was run in Minitab^®^, and an experiment of 156 samples (52 trials per 3 blocks) was obtained and performed independently. The design is presented in S1 Table.

- **Characterization of AuNR**

**UV–Vis spectroscopy:** The optical properties of the gold nanostructures were determined using a UV–Vis spectrophotometer (Multiskan GO, Thermo Scientific) to obtain the surface plasmon bands between 400 and 600 nm and the full width at half maximum factor (FWHM). Two hundred and fifty microliters of the samples were added in triplicate at 37 °C in a 96-well microplate to obtain the absorbance of the nanostructures.

**Transmission electron microscopy (TEM):** The morphology and size of the nanostructures were determined using the TEM technique using a Tecnai F20 Super Twin TMP microscope (FEI). For this assay, 20 μL of the samples were served on a copper grid previously coated with Formvar (Sigma-Aldrich).

## 3. Results and discussions

### 3.1 Obtention of *Psidium araca* extract

**Fig 1:**
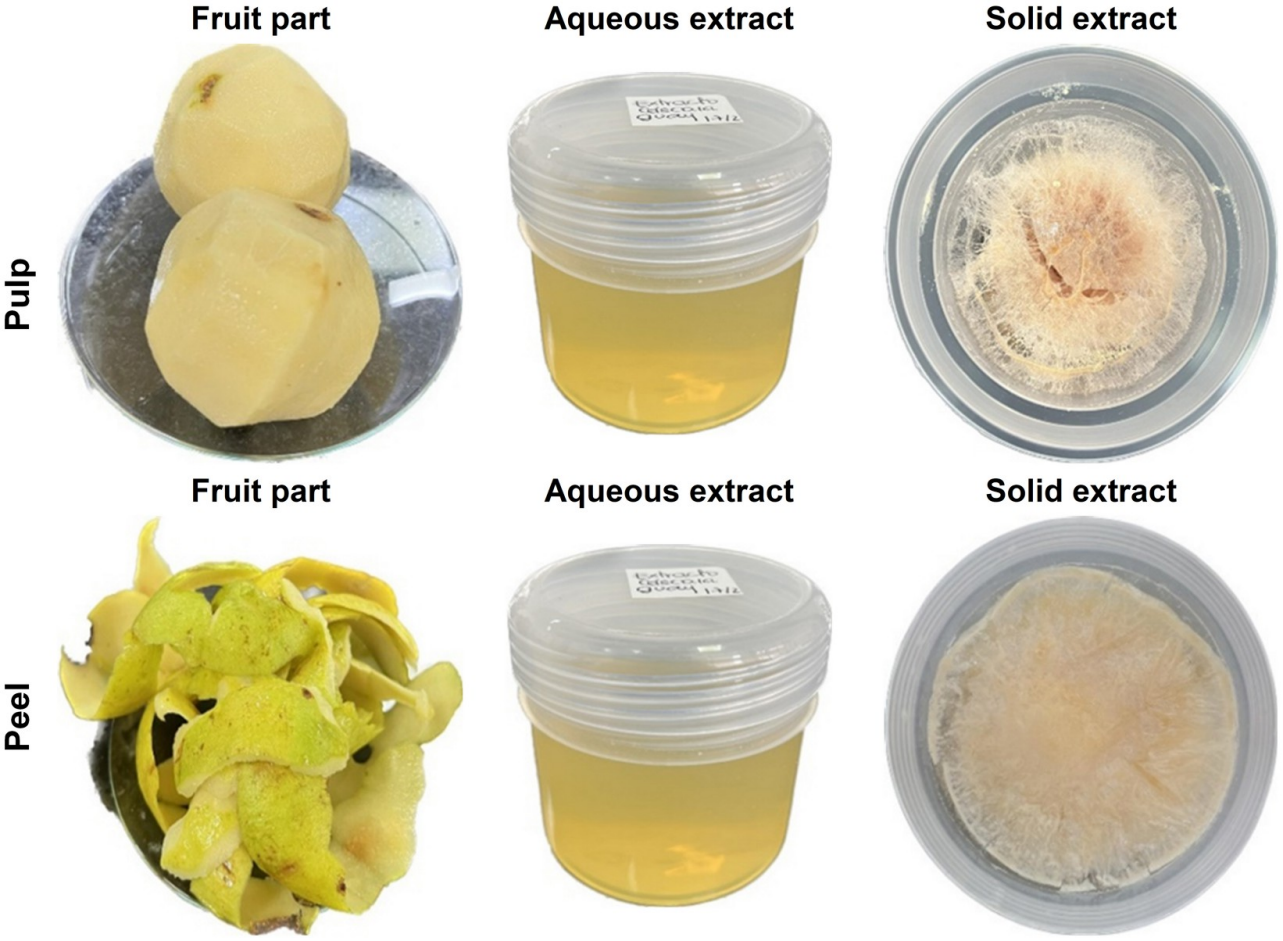
Aqueous and solid extracts of pulp and peel sour guava.

Fig 1 shows the preparation process of the sour guava pulp and peel extracts. Initially, the whole fruit was collected and separated into the pulp and peel. After obtaining the aqueous extract, the fruit was lyophilized and solid extracts were obtained to preserve the chemical properties of each fruit from environmental factors such as humidity, temperature, and light.

### 3.2 Total phenols and antioxidant capacity of *Psidium araca* extract over time

The colorimetric techniques and the equations mentioned in section 2.3 were used to select the guava part with the best antioxidant properties between the pulp and peel extracts over 0, 7, and 14 days. Table 3 shows the total phenol content and antioxidant capacity (ABTS, FRAP, and DPPH) at 0, 7, and 14 days.

**Table 3:** Results of the study of the antioxidant capacity over time of an aqueous extract of pulp and peel of sour guava.

Assay*	Extract	Day 0	Day 7	Day 14
**Total phenol** **(mg gallic acid (ga)/100 g)**	Pulp	515.5 ± 193.8	422.8 ± 14.5	481.3 ± 29.4
	Peel	575.8 ± 22.8	575.8 ± 546.9	735.3 ± 47.4
**ABTS** **(μmol Trolox/100 g)**	Pulp	15,884.1 ± 667.2	13,842.8 ± 1,586.9	8,624.7 ± 787.5
	Peel	14,654.86 ± 2,378.04	14,508.1 ± 1,385.8	9,923.1 ± 2,032.6
**FRAP** (**Fe**^**2+**^ **nmol/g**)	Pulp	37.1 ± 20.9	71.9 ± 15.1	9.2 ± 1.3
	Peel	67.8 ± 22.2	76.4 ± 28.5	38.6 ± 3.3
**DPPH** **(EC50) (mg/mL)**	Pulp	0.0218	0.0303	0.0249
	Peel	0.0005	0.0180	4.738 x 10^−5^

*All assays were performed in triplicate except for the DPPH assay.

Because the antioxidant capacity results were not comparable because each used different standard reference molecules [24], statistical analysis was performed to compare the variations in time for each test. Nonetheless, it was observed that the sour guava peel extract exhibited the highest concentration of total phenols and antioxidant activity, evaluated by the FRAP and DPPH techniques from day 0 with values of 575.8 ± 22.8 mg ga/100 g, 67.8 ± 22.2 nmol Fe^2+^/g and 0.0005 mg/mL, respectively. By contrast, in the ABTS assay, the pulp extract presented a greater average antioxidant capacity on day 0. However, on days 7 and 14, the peel extract exhibited the highest antioxidant activity. Owing to the nature of the ABTS radical, which is reduced in the presence of antioxidant molecules, an ambiguous effect can be generated when evaluating hydrophilic and lipophilic molecules [24].

Statistical analysis of the amount of total phenols in the pulp and peel extracts over time revealed statistically significant differences (p = 0.033), indicating that the peel had the greatest antioxidant capacity over time. This result confirms that in the design analysis, the shell section presented a higher concentration of total phenols during storage kinetics. Statistical analysis also indicated significant differences for the time factor (days of storage of the extract in solid state) with a p-value < 0.001; therefore, it can be concluded that the antioxidant properties of the ABTS assay decreased from day 14 onward, as seen in the time trend in Table 3. Finally, using the FRAP assay, we found that the fruit section and day presented significant differences (p = 0.031 and 0.003, respectively). In summary, considering previous results, sour guava peel extract was selected as a weak reducing agent for AuNR biosynthesis, and the solid extract was stored for a maximum of 7 days after freeze-drying.

### 3.3 Synthesis and characterization of AuNRs by the seedless method using *Psidium araca* extract

From the experimental design used to obtain AuNRs by biosynthesis with guava peel extract, it was found that out of 52 runs, only AuNRs were obtained in six experiments (in triplicate). Table 4 shows the experimental conditions and output variables (LSPR) for each AuNR assay with its replicates and photographic records used to identify the characteristic colors of the AuNR dispersion.

**Table 4:** Experimental conditions and output variable (LSPR) *and color* of each assay that formed the AuNRs.

Sample	Run*	Time (h)	HAuCl_4_•3H_2_O (mM)	AgNO_3_ (mM)	Extract (mg/mL)	NaBH_4_ (mM)	LSPR (nm)	LSPR (nm) Average ± SD
1	30	48	60	30	30	10	795	769.7 ± 41.3
1	56	48	60	30	30	10	722	
1	138	48	60	30	30	10	792	
2	111	24	60	30	30	10	813	798.0 ± 13.8
2	10	24	60	30	30	10	795	
2	72	24	60	30	30	10	786	
3	7	48	40	30	30	10	778	800.7 ± 20.5
3	90	48	40	30	30	10	806	
3	136	48	40	30	30	10	818	
4	13	24	40	30	30	10	771	750.8 ± 18.4
4	74	24	40	30	30	10	746	
4	107	24	40	30	30	10	735	
5	28	24	40	10	30	10	773	766.3 ± 5.8
5	65	24	40	10	30	10	760	
5	137	24	40	10	30	10	760	
6	96	24	60	10	30	10	708	715.3 ± 6.4
6	41	24	60	10	30	10	719	
6	150	24	60	10	30	10	719	

* Numbers in the experimental design

**Fig 2:**
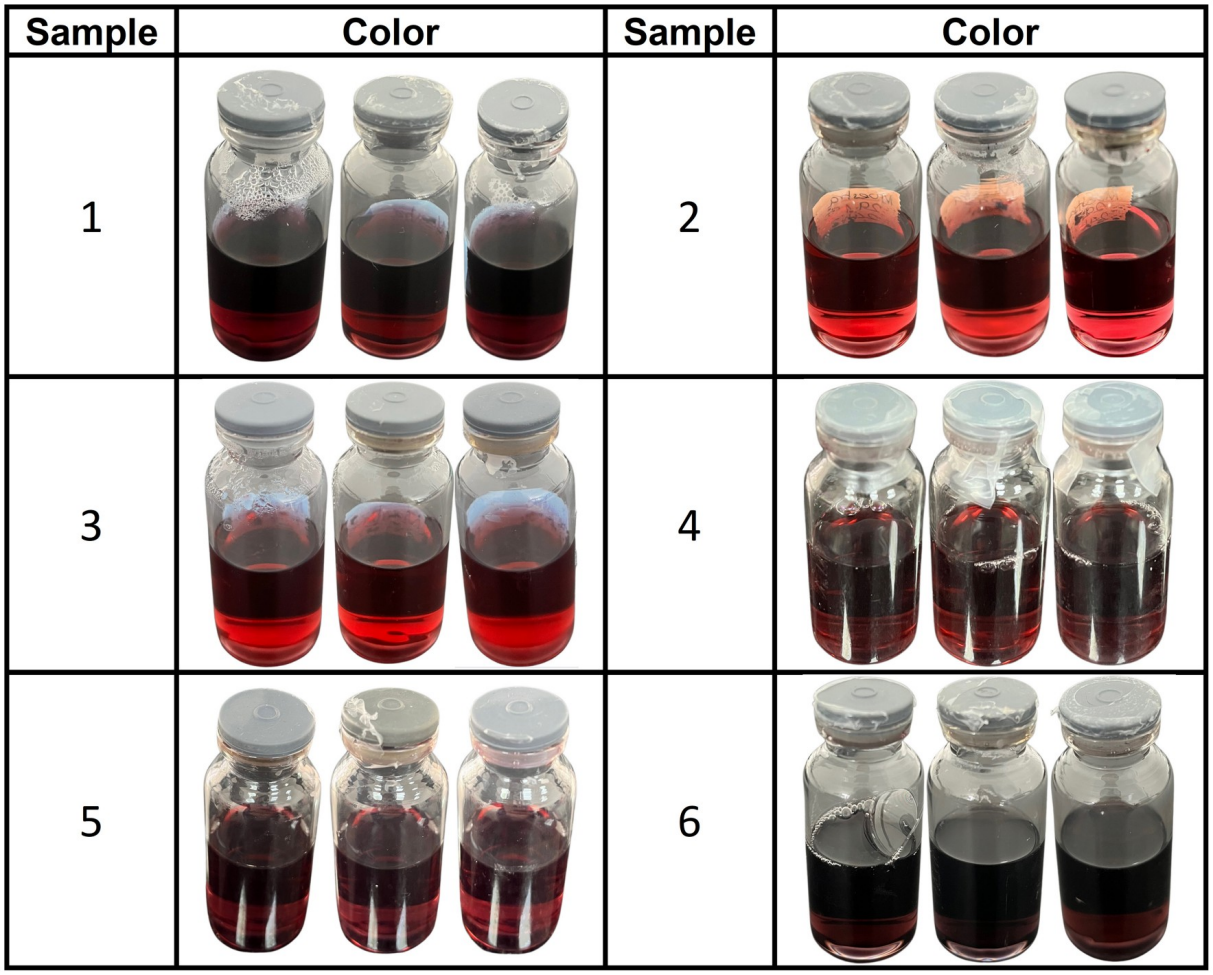
Photography of the dispersion that shows the red wine color characteristic of gold nanorods.

The distinct red wine color associated with this morphology was evident [1] (Fig 2), which arises because of the interaction between the incident light reflected on the dispersions in the presence of AuNRs [26]. However, the variability in color tone and intensity was attributed to the morphological variations that occur in the aspect ratio of AuNRs [27].

The morphology was also corroborated by the two surface plasmon bands between 400 and 1000 nm found in the UV–Vis spectra of all the samples, as shown in S1 Fig. The transverse band (TSPR) occurred at 538–548 nm, whereas the longitudinal band (LSPR) occurred at 669–818 nm. By contrast, the absorbance intensity of each resonance band was associated with the purity of the dispersion (Table 5) [28], revealing the heterogeneity of the samples.

**Table 5:** Absorbance intensity of surface plasmon bands (TSPR and LSPR).

Sample*	Absorbance intensity in TSPR	Absorbance intensity in LSPR
1	0.54 ± 0.02	0.36 ± 0.05
2	0.58 ± 0.01	0.31 ± 0.03
3	0.57 ± 0.05	0.31 ± 0.03
4	0.57 ± 0.03	0.27 ± 0.01
5	0.53 ± 0.02	0.31 ± 0.05
6	0.40 ± 0.01	0.60 ± 0.05

*All assays were performed in triplicate

Considering that the intensity of the transverse bands (TSPR) is higher than the intensity of the absorbances of the longitudinal bands (LSPR), it can be inferred that the dispersions contain a high concentration of spherical or cubic nanoparticles, as other studies have reported that the spheres absorb between 522 and 570 nm [29], where the TSPR of the bars also absorbs. These results suggest polydispersity in the samples; hence, full width at half maximum (FWHM) values were calculated for the transverse and longitudinal bands of each spectrum [30]. The results are presented in Table 6.

**Table 6:** FWHM values of the transverse (TSPR) and longitudinal (LSPR) bands.

Sample*	FWHM	
	TSPR	LSPR
1	72.6 ±1.1	157.7 ± 11.2
2	75.0 ± 1.1	165.3 ± 20.4
3	62.6 ± 2.0	158.1 ± 6.8
4	74.4 ± 1.3	146.5 ± 9.1
5	72.3 ± 2.7	159.7 ± 1.3
6	67.5 ± 1.3	124.9 ± 8.3

*All assays were performed in triplicate 3

Based on the definition of the FWHM [31, 32] parameter, nanoparticles such as AuNRs generally have a lower FWHM in the transverse area than in the longitudinal area owing to the anisotropy presented in this morphology [32], which was identified for all samples. Additionally, because a lower FWHM value indicates less polydispersity [31], sample 6 had a lower FWHM factor in the longitudinal band (LSPR) than the other samples, suggesting that there were bars with more homogeneous longitudinal sizes.

**Fig 3:**
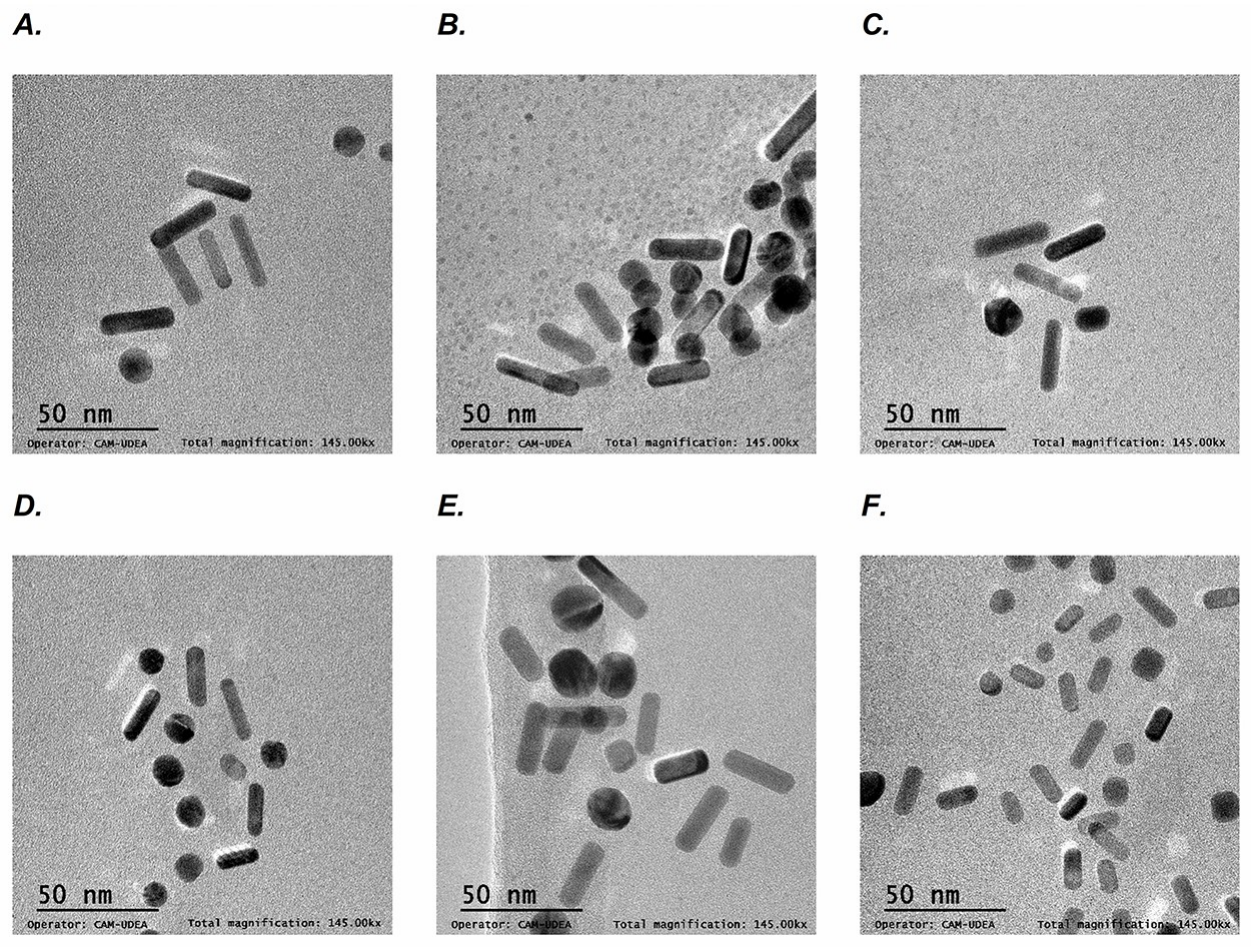
TEM images A. Sample 1, B. Sample 2, C. Sample 3, D. Sample 4, E. Sample 5, and F. Sample 6.

In agreement with the UV–Vis spectral analysis, the TEM images confirmed the presence of different types of morphologies in the analyzed dispersions (Fig 3), with sizes of approximately 30 nm for all samples. In addition to nanorods, seeds, cubes, and nanospheres were found in all dispersions; therefore, the purification parameters were adjusted to increase the population of nanorods in the dispersions [33]. However, other purification methods need to be explored in future studies.

A response surface experimental design was selected to optimize the synthesis parameters with the sour guava extract, and the interactions between the factors used in the synthesis of AuNRs were evaluated to control the morphology and size of the nanorod to obtain an LSPR of 808 nm. The results of the response surface statistical model presented a fit with R^2^ = 77.09%. After analyzing the details of the samples that gave an LSPR close to the target value, the significances of the factors evaluated were determined using *Minitab*^*®*^ (Table 7). Eq 7 shows the obtained design equation.


$$\begin{array}{l} \begin{array}{l} LSPR=233-1.0\;\text{Oro}+5.10\;\text{Extract}-0.1955\;\text{Time}*\text{Time}-0.476\\ \text{Silver}*\text{Silver}-0.1632\;\text{Time}*\text{Gold}+0.2454\;\text{Gold}*\text{Silver} \end{array} \end{array}$$

**Table 7:** Significant differences from the response surface experimental design.

**Linear factor**	***P value***
Gold (HAuCl_4_•3H_2_O)	0.014
Extract	<0.001
**Interaction of factors**	***P value***
Time*Time	0.013
Silver*Silver (AgNO_3_)	0.009
Time*Gold	0.013
Gold*Silver	0.004

Based on these results, the gold and extract factors were linear and significant (p = 0.014 and p > 0.001, respectively). Therefore, by varying the concentrations of the linear factors of gold and the extract, the growth of AuNRs could be controlled to obtain the longitudinal plasmon of interest.

**Fig 4:**
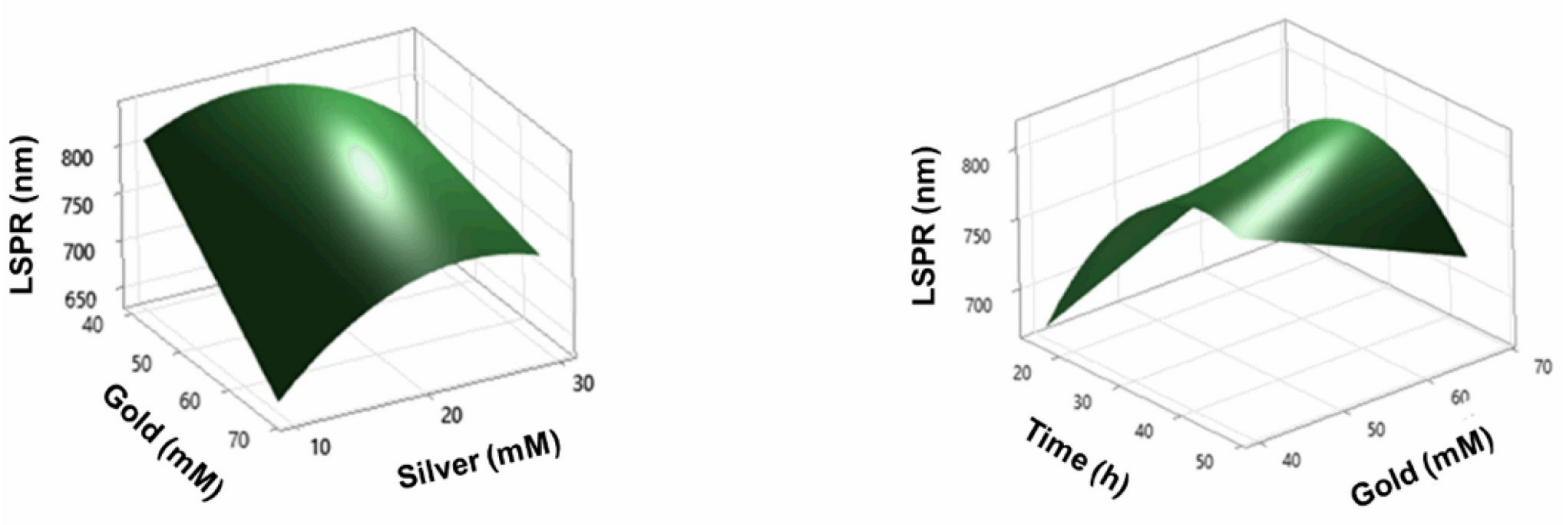
Response surfaces of the experimental design.

**Fig 5:**
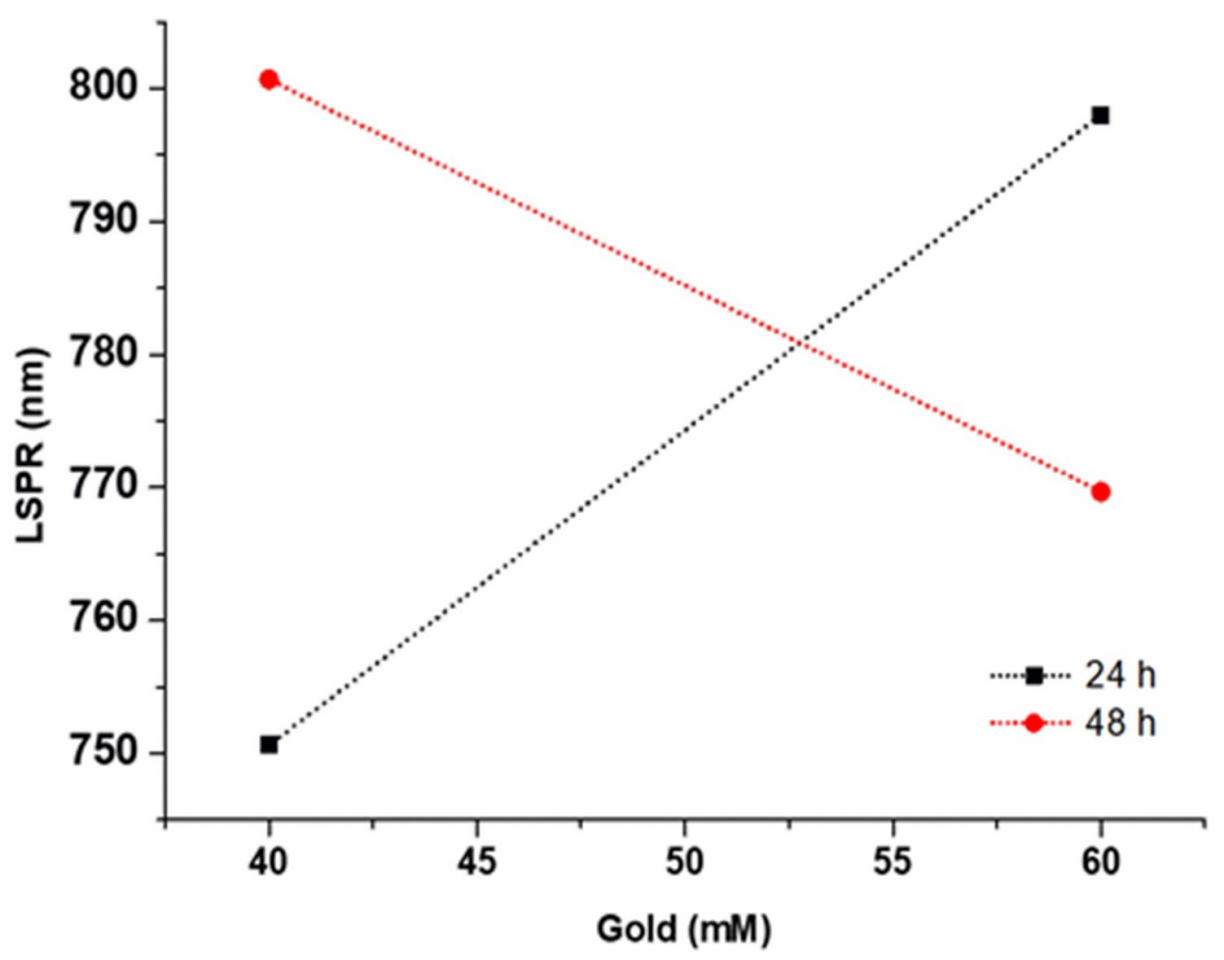
Variation of the LSPR with the parameters time and gold concentration.

Similarly, interactions were found between some factors with statistically significant differences, as shown in Table 7; consequently, two response surfaces were found (Fig 4). One of these interactions was the relationship between time and the amount of gold, as there must be a compensation between these two variables, keeping the other variables fixed at 30 mM silver, 30 mg/mL extract, and 10 mM NaBH_4_. Therefore, when the factor time was 24 h and the amount of gold increased, the bar size also increased. However, when the reaction time was longer (48 h) and the amount of gold increased, the size of the bars decreased (Fig 5).

However, an effect was also evident concerning the amount of silver nitrate; in this case, increasing the concentration of silver nitrate decreased the length of the bar length. Although a gradual increase in the concentration of Ag^+^ ions leads to bar growth, this is still being debated because of the multiple mechanisms through which Ag^+^ ions can be deposited on gold bars, affecting their growth [3]. Three mechanisms involving Ag^+^ ions can modulate rod growth: 1) underpotential deposition, 2) formation of an Ag[BrCTA]_2_ complex that explicitly covers the lateral faces of the growing seeds, and 3) modification of CTAB micelle formation through AgBr interactions [3].

Specifically, adding AgNO_3_ to a CTAB solution leads to the immediate formation of AgBr; therefore, this species plays an essential role in the formation mechanism of AuNRs because Ag^+^ ions are absorbed on the surface of gold particles in the form of AgBr, which restricts their growth [34].

Regarding the amount of extract, a decrease in the amount of extract led to a reduction in the length of the rod, which could be related to the antioxidant properties of the extract. A lower concentration of this substance generates a weak reducing effect and fewer molecules exert this effect. Therefore, fewer reduced gold ions could be deposited at the nucleation points, leading to less bar growth.

Finally, with borohydride, a strong reducing agent, it was evident that by increasing the amount of this reagent and keeping the other variables constant, there was a decrease in the longitudinal plasmon resonance (LSPR), which may be related to the possibility of increasing the nucleation points by increasing the borohydride concentration. Therefore, more nuclei were formed, but the rods grew less when the time remained constant. However, if the concentration was increased and the time was also increased (from 24 to 48 h), the LSPR increased, indicating that, although the nucleation centers were increased, there was a longer time for these centers to increase their size.

The optimal response was sought to obtain nanorods with an LSPR of 808 nm using the information obtained from the response surface. The optimized factors are shown in Table 8 with a 95% confidence interval (CI) = (771.1 nm; 844.9 nm) and a 95% prediction interval (PI) = (744.6 nm; 871.4 nm). The optimal response of the model was verified experimentally using the factors listed in Table 8.

**Table 8:** Factors of the optimal response of the model (LSPR = 808 nm).

Factor	Level
Time (h)	46.58
Gold (HAuCl_4_•3H_2_O) (mM)	40
Silver (AgNO_3_) (mM)	25.22
Extract (mg/mL)	21.87
NaBH_4_ (mM)	10

**Fig 6:**
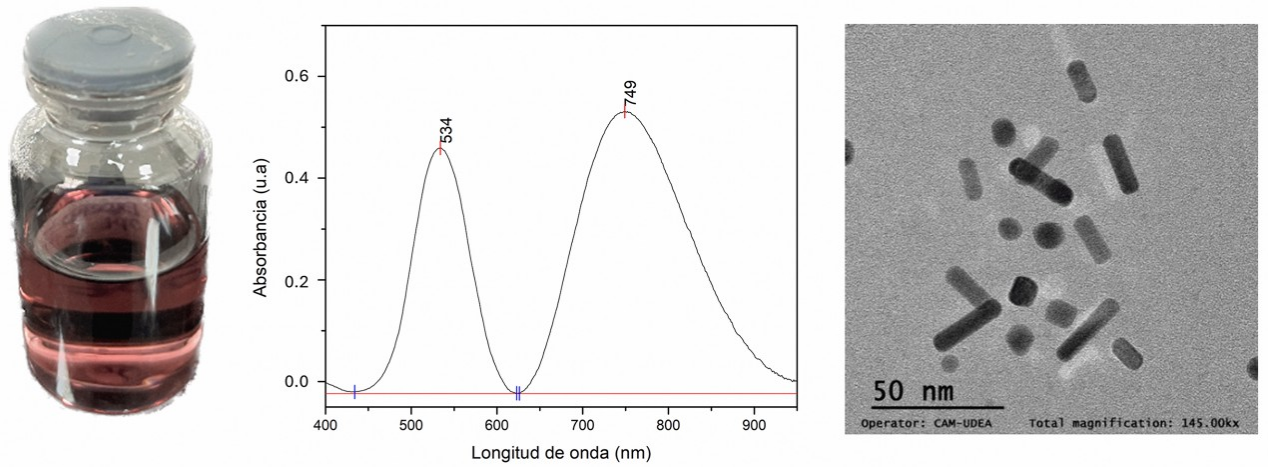
Optical and morphological properties: Color and UV–Vis spectrum and TEM results.

The experimental results are presented in Fig 6. The dispersion was light purple, indicating the presence of Au nanostructures in the sample. Two surface plasmon bands were identified by analyzing the optical properties of the UV–Vis spectrum. The first band was observed at 534 nm, which was attributed to the cross-section of the bars and the presence of other types of morphologies, such as spheres and cubes [35]. A second band was observed at 749 nm, indicating the formation of AuNRs. The TEM images confirmed the presence of nanorods, nanospheres, and nanocubes in the dispersion.

Given that the LSPR was presented at 749 nm, this falls within the prediction range established by the model, which considers the percentage of variability that can occur when examining the interaction between the factors. However, because there is no response at 808 nm, some unknown information remains regarding the possible interaction between traditional factors (gold, silver, and borohydride) and the aqueous extract of sour guava because this green agent contains different chemical components in its structure [15], which can vary the conditions for the formation of AuNRs.

## 4. Conclusions

An aqueous extract of sour guava (*Psidium araca*) was successfully used as a new green weakly reducing agent for the biosynthesis of gold nanorods. Specifically, the aqueous peel extract showed the best antioxidant capacity and was selected to control different parameters of the synthesis of AuNRs, such as morphology and LSPR. In addition, the solid extract could be stored for up to 7 days after freeze-drying because, after this time, the antioxidant capacity decreased.

Response surface experimental designs were used to establish a protocol for obtaining AuNRs with LSPR close to 808 nm, considering the synthesis time and concentrations of gold, silver, extract, and sodium borohydride. From the experimental design, it was found that it was possible to obtain AuNRs with the required surface plasmons close to 808 nm (samples 2 and 3) using the conditions summarized in Table 9. Furthermore, by varying the concentrations of the linear factors of gold and the extract, the growth of AuNRs could be controlled to obtain a longitudinal plasmon of interest. Similarly, the interaction between gold factors influences the synthesis of AuNRs, indicating that variations in the concentrations of these factors can alter their morphology and size. Although the gold–silver factor also had interactions, and consequently, there was a dependence between them, the effect of this dependence must be analyzed in the context of other reactants. Finally, the percentage variability in the optimal response obtained using the experimental design can be attributed to the unknown chemical composition of the green extract.

**Table 9:** Summary of conditions to synthesize AuNRs using the aqueous extract of peeled sour guava.

Time (h)	HAuCl_4_•3H_2_O (mM)	AgNO_3_ (mM)	Extract (mg/mL)	NaBH_4_ (mM)
24	60	30	30	10
48	40	30	30	10

## Supporting information

S1 Table Response surface experimental design. (DOCX)

S1 Fig UV-Vis spectra of all AuNR synthetized. (DOCX)
