# Supplementary material for: Optimization of the green synthesis of gold nanorods using aqueous extract of peeled sour guava as a source of antioxidants
Source: PLoS One. 2025 Jan 8;20(1):e0313485. doi: 10.1371/journal.pone.0313485 (PMC11709274; doi:10.1371/journal.pone.0313485)
Supplement: S1 Table — (DOCX) [file pone.0313485.s001.docx]

**Supporting Information**

**S1 Table.** Response surface experimental design

| OrdenEst | Run | TypePt | Blocks | Time (h) | Gold (mM) | Plata (mM) | Extracto (mg/mL) | NaBH_4_ (mM) |
| --- | --- | --- | --- | --- | --- | --- | --- | --- |
| 156 | 1 | 0 | 3 | 36 | 50 | 20 | 20 | 6.5 |
| 145 | 2 | -1 | 3 | 36 | 50 | 20 | 20 | 0.55 |
| 112 | 3 | 1 | 3 | 48 | 60 | 30 | 10 | 3 |
| 147 | 4 | 0 | 3 | 36 | 50 | 20 | 20 | 6.5 |
| 113 | 5 | 1 | 3 | 24 | 40 | 10 | 30 | 3 |
| 143 | 6 | -1 | 3 | 36 | 50 | 20 | 3 | 6.5 |
| 134 | 7 | 1 | 3 | 48 | 40 | 30 | 30 | 10 |
| 115 | 8 | 1 | 3 | 24 | 60 | 10 | 30 | 3 |
| 152 | 9 | 0 | 3 | 36 | 50 | 20 | 20 | 6.5 |
| 135 | 10 | 1 | 3 | 24 | 60 | 30 | 30 | 10 |
| 111 | 11 | 1 | 3 | 24 | 60 | 30 | 10 | 3 |
| 123 | 12 | 1 | 3 | 24 | 60 | 10 | 10 | 10 |
| 133 | 13 | 1 | 3 | 24 | 40 | 30 | 30 | 10 |
| 125 | 14 | 1 | 3 | 24 | 40 | 30 | 10 | 10 |
| 141 | 15 | -1 | 3 | 36 | 50 | 3 | 20 | 6.5 |
| 109 | 16 | 1 | 3 | 24 | 40 | 30 | 10 | 3 |
| 140 | 17 | -1 | 3 | 36 | 67 | 20 | 20 | 6.5 |
| 139 | 18 | -1 | 3 | 36 | 33 | 20 | 20 | 6.5 |
| 144 | 19 | -1 | 3 | 36 | 50 | 20 | 37 | 6.5 |
| 118 | 20 | 1 | 3 | 48 | 40 | 30 | 30 | 3 |
| 155 | 21 | 0 | 3 | 36 | 50 | 20 | 20 | 6.5 |
| 126 | 22 | 1 | 3 | 48 | 40 | 30 | 10 | 10 |
| 138 | 23 | -1 | 3 | 56.4 | 50 | 20 | 20 | 6.5 |
| 149 | 24 | 0 | 3 | 36 | 50 | 20 | 20 | 6.5 |
| 146 | 25 | -1 | 3 | 36 | 50 | 20 | 20 | 12.45 |
| 132 | 26 | 1 | 3 | 48 | 60 | 10 | 30 | 10 |
| 153 | 27 | 0 | 3 | 36 | 50 | 20 | 20 | 6.5 |
| 129 | 28 | 1 | 3 | 24 | 40 | 10 | 30 | 10 |
| 151 | 29 | 0 | 3 | 36 | 50 | 20 | 20 | 6.5 |
| 136 | 30 | 1 | 3 | 48 | 60 | 30 | 30 | 10 |
| 128 | 31 | 1 | 3 | 48 | 60 | 30 | 10 | 10 |
| 154 | 32 | 0 | 3 | 36 | 50 | 20 | 20 | 6.5 |
| 120 | 33 | 1 | 3 | 48 | 60 | 30 | 30 | 3 |
| 119 | 34 | 1 | 3 | 24 | 60 | 30 | 30 | 3 |
| 137 | 35 | -1 | 3 | 15.6 | 50 | 20 | 20 | 6.5 |
| 107 | 36 | 1 | 3 | 24 | 60 | 10 | 10 | 3 |
| 114 | 37 | 1 | 3 | 48 | 40 | 10 | 30 | 3 |
| 122 | 38 | 1 | 3 | 48 | 40 | 10 | 10 | 10 |
| 121 | 39 | 1 | 3 | 24 | 40 | 10 | 10 | 10 |
| 130 | 40 | 1 | 3 | 48 | 40 | 10 | 30 | 10 |
| 131 | 41 | 1 | 3 | 24 | 60 | 10 | 30 | 10 |
| 150 | 42 | 0 | 3 | 36 | 50 | 20 | 20 | 6.5 |
| 124 | 43 | 1 | 3 | 48 | 60 | 10 | 10 | 10 |
| 148 | 44 | 0 | 3 | 36 | 50 | 20 | 20 | 6.5 |
| 127 | 45 | 1 | 3 | 24 | 60 | 30 | 10 | 10 |
| 116 | 46 | 1 | 3 | 48 | 60 | 10 | 30 | 3 |
| 110 | 47 | 1 | 3 | 48 | 40 | 30 | 10 | 3 |
| 105 | 48 | 1 | 3 | 24 | 40 | 10 | 10 | 3 |
| 117 | 49 | 1 | 3 | 24 | 40 | 30 | 30 | 3 |
| 106 | 50 | 1 | 3 | 48 | 40 | 10 | 10 | 3 |
| 108 | 51 | 1 | 3 | 48 | 60 | 10 | 10 | 3 |
| 142 | 52 | -1 | 3 | 36 | 50 | 37 | 20 | 6.5 |
| 48 | 53 | 0 | 1 | 36 | 50 | 20 | 20 | 6.5 |
| 13 | 54 | 1 | 1 | 24 | 40 | 30 | 30 | 3 |
| 43 | 55 | 0 | 1 | 36 | 50 | 20 | 20 | 6.5 |
| 32 | 56 | 1 | 1 | 48 | 60 | 30 | 30 | 10 |
| 4 | 57 | 1 | 1 | 48 | 60 | 10 | 10 | 3 |
| 8 | 58 | 1 | 1 | 48 | 60 | 30 | 10 | 3 |
| 33 | 59 | -1 | 1 | 15.6 | 50 | 20 | 20 | 6.5 |
| 12 | 60 | 1 | 1 | 48 | 60 | 10 | 30 | 3 |
| 3 | 61 | 1 | 1 | 24 | 60 | 10 | 10 | 3 |
| 9 | 62 | 1 | 1 | 24 | 40 | 10 | 30 | 3 |
| 40 | 63 | -1 | 1 | 36 | 50 | 20 | 37 | 6.5 |
| 42 | 64 | -1 | 1 | 36 | 50 | 20 | 20 | 12.45 |
| 25 | 65 | 1 | 1 | 24 | 40 | 10 | 30 | 10 |
| 39 | 66 | -1 | 1 | 36 | 50 | 20 | 3 | 6.5 |
| 18 | 67 | 1 | 1 | 48 | 40 | 10 | 10 | 10 |
| 41 | 68 | -1 | 1 | 36 | 50 | 20 | 20 | 0.55 |
| 22 | 69 | 1 | 1 | 48 | 40 | 30 | 10 | 10 |
| 6 | 70 | 1 | 1 | 48 | 40 | 30 | 10 | 3 |
| 21 | 71 | 1 | 1 | 24 | 40 | 30 | 10 | 10 |
| 31 | 72 | 1 | 1 | 24 | 60 | 30 | 30 | 10 |
| 26 | 73 | 1 | 1 | 48 | 40 | 10 | 30 | 10 |
| 29 | 74 | 1 | 1 | 24 | 40 | 30 | 30 | 10 |
| 35 | 75 | -1 | 1 | 36 | 33 | 20 | 20 | 6.5 |
| 17 | 76 | 1 | 1 | 24 | 40 | 10 | 10 | 10 |
| 7 | 77 | 1 | 1 | 24 | 60 | 30 | 10 | 3 |
| 52 | 78 | 0 | 1 | 36 | 50 | 20 | 20 | 6.5 |
| 23 | 79 | 1 | 1 | 24 | 60 | 30 | 10 | 10 |
| 38 | 80 | -1 | 1 | 36 | 50 | 37 | 20 | 6.5 |
| 1 | 81 | 1 | 1 | 24 | 40 | 10 | 10 | 3 |
| 36 | 82 | -1 | 1 | 36 | 67 | 20 | 20 | 6.5 |
| 50 | 83 | 0 | 1 | 36 | 50 | 20 | 20 | 6.5 |
| 28 | 84 | 1 | 1 | 48 | 60 | 10 | 30 | 10 |
| 46 | 85 | 0 | 1 | 36 | 50 | 20 | 20 | 6.5 |
| 11 | 86 | 1 | 1 | 24 | 60 | 10 | 30 | 3 |
| 51 | 87 | 0 | 1 | 36 | 50 | 20 | 20 | 6.5 |
| 15 | 88 | 1 | 1 | 24 | 60 | 30 | 30 | 3 |
| 44 | 89 | 0 | 1 | 36 | 50 | 20 | 20 | 6.5 |
| 30 | 90 | 1 | 1 | 48 | 40 | 30 | 30 | 10 |
| 37 | 91 | -1 | 1 | 36 | 50 | 3 | 20 | 6.5 |
| 45 | 92 | 0 | 1 | 36 | 50 | 20 | 20 | 6.5 |
| 20 | 93 | 1 | 1 | 48 | 60 | 10 | 10 | 10 |
| 47 | 94 | 0 | 1 | 36 | 50 | 20 | 20 | 6.5 |
| 19 | 95 | 1 | 1 | 24 | 60 | 10 | 10 | 10 |
| 27 | 96 | 1 | 1 | 24 | 60 | 10 | 30 | 10 |
| 34 | 97 | -1 | 1 | 56.4 | 50 | 20 | 20 | 6.5 |
| 24 | 98 | 1 | 1 | 48 | 60 | 30 | 10 | 10 |
| 14 | 99 | 1 | 1 | 48 | 40 | 30 | 30 | 3 |
| 16 | 100 | 1 | 1 | 48 | 60 | 30 | 30 | 3 |
| 49 | 101 | 0 | 1 | 36 | 50 | 20 | 20 | 6.5 |
| 5 | 102 | 1 | 1 | 24 | 40 | 30 | 10 | 3 |
| 2 | 103 | 1 | 1 | 48 | 40 | 10 | 10 | 3 |
| 10 | 104 | 1 | 1 | 48 | 40 | 10 | 30 | 3 |
| 93 | 105 | -1 | 2 | 36 | 50 | 20 | 20 | 0.55 |
| 61 | 106 | 1 | 2 | 24 | 40 | 10 | 30 | 3 |
| 81 | 107 | 1 | 2 | 24 | 40 | 30 | 30 | 10 |
| 78 | 108 | 1 | 2 | 48 | 40 | 10 | 30 | 10 |
| 75 | 109 | 1 | 2 | 24 | 60 | 30 | 10 | 10 |
| 102 | 110 | 0 | 2 | 36 | 50 | 20 | 20 | 6.5 |
| 83 | 111 | 1 | 2 | 24 | 60 | 30 | 30 | 10 |
| 63 | 112 | 1 | 2 | 24 | 60 | 10 | 30 | 3 |
| 76 | 113 | 1 | 2 | 48 | 60 | 30 | 10 | 10 |
| 62 | 114 | 1 | 2 | 48 | 40 | 10 | 30 | 3 |
| 97 | 115 | 0 | 2 | 36 | 50 | 20 | 20 | 6.5 |
| 99 | 116 | 0 | 2 | 36 | 50 | 20 | 20 | 6.5 |
| 70 | 117 | 1 | 2 | 48 | 40 | 10 | 10 | 10 |
| 87 | 118 | -1 | 2 | 36 | 33 | 20 | 20 | 6.5 |
| 58 | 119 | 1 | 2 | 48 | 40 | 30 | 10 | 3 |
| 95 | 120 | 0 | 2 | 36 | 50 | 20 | 20 | 6.5 |
| 94 | 121 | -1 | 2 | 36 | 50 | 20 | 20 | 12.45 |
| 74 | 122 | 1 | 2 | 48 | 40 | 30 | 10 | 10 |
| 55 | 123 | 1 | 2 | 24 | 60 | 10 | 10 | 3 |
| 90 | 124 | -1 | 2 | 36 | 50 | 37 | 20 | 6.5 |
| 53 | 125 | 1 | 2 | 24 | 40 | 10 | 10 | 3 |
| 91 | 126 | -1 | 2 | 36 | 50 | 20 | 3 | 6.5 |
| 67 | 127 | 1 | 2 | 24 | 60 | 30 | 30 | 3 |
| 69 | 128 | 1 | 2 | 24 | 40 | 10 | 10 | 10 |
| 65 | 129 | 1 | 2 | 24 | 40 | 30 | 30 | 3 |
| 98 | 130 | 0 | 2 | 36 | 50 | 20 | 20 | 6.5 |
| 101 | 131 | 0 | 2 | 36 | 50 | 20 | 20 | 6.5 |
| 103 | 132 | 0 | 2 | 36 | 50 | 20 | 20 | 6.5 |
| 86 | 133 | -1 | 2 | 56.4 | 50 | 20 | 20 | 6.5 |
| 72 | 134 | 1 | 2 | 48 | 60 | 10 | 10 | 10 |
| 100 | 135 | 0 | 2 | 36 | 50 | 20 | 20 | 6.5 |
| 82 | 136 | 1 | 2 | 48 | 40 | 30 | 30 | 10 |
| 77 | 137 | 1 | 2 | 24 | 40 | 10 | 30 | 10 |
| 84 | 138 | 1 | 2 | 48 | 60 | 30 | 30 | 10 |
| 92 | 139 | -1 | 2 | 36 | 50 | 20 | 37 | 6.5 |
| 96 | 140 | 0 | 2 | 36 | 50 | 20 | 20 | 6.5 |
| 85 | 141 | -1 | 2 | 15.6 | 50 | 20 | 20 | 6.5 |
| 57 | 142 | 1 | 2 | 24 | 40 | 30 | 10 | 3 |
| 60 | 143 | 1 | 2 | 48 | 60 | 30 | 10 | 3 |
| 73 | 144 | 1 | 2 | 24 | 40 | 30 | 10 | 10 |
| 104 | 145 | 0 | 2 | 36 | 50 | 20 | 20 | 6.5 |
| 68 | 146 | 1 | 2 | 48 | 60 | 30 | 30 | 3 |
| 80 | 147 | 1 | 2 | 48 | 60 | 10 | 30 | 10 |
| 88 | 148 | -1 | 2 | 36 | 67 | 20 | 20 | 6.5 |
| 56 | 149 | 1 | 2 | 48 | 60 | 10 | 10 | 3 |
| 79 | 150 | 1 | 2 | 24 | 60 | 10 | 30 | 10 |
| 71 | 151 | 1 | 2 | 24 | 60 | 10 | 10 | 10 |
| 89 | 152 | -1 | 2 | 36 | 50 | 3 | 20 | 6.5 |
| 66 | 153 | 1 | 2 | 48 | 40 | 30 | 30 | 3 |
| 54 | 154 | 1 | 2 | 48 | 40 | 10 | 10 | 3 |
| 64 | 155 | 1 | 2 | 48 | 60 | 10 | 30 | 3 |
| 59 | 156 | 1 | 2 | 24 | 60 | 30 | 10 | 3 |
