# Supplementary figures and images for: Optimization of the green synthesis of gold nanorods using aqueous extract of peeled sour guava as a source of antioxidants
Source: PLoS One. 2025 Jan 8;20(1):e0313485. doi: 10.1371/journal.pone.0313485 (PMC11709274; doi:10.1371/journal.pone.0313485)

**Supporting Information**

**S1 Fig.** UV-Vis spectra of all AuNR synthesized

| A. | B. | C. |
| --- | --- | --- |
| 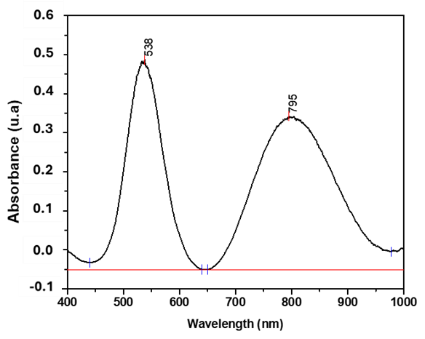 | 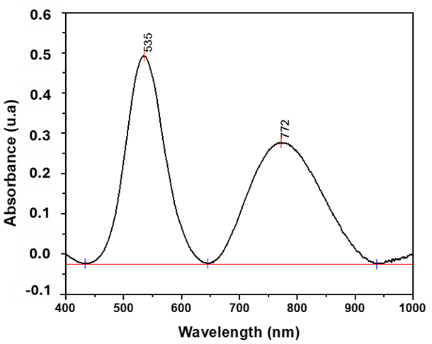 | 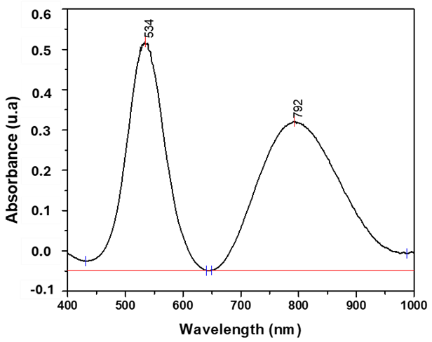 |
| D. | E. | F. |
| 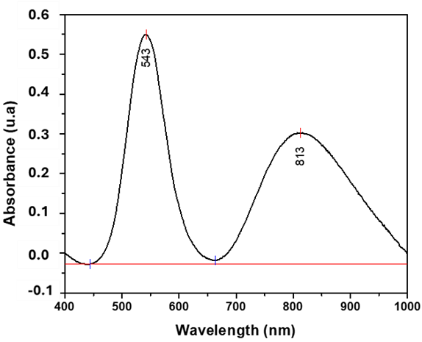 | 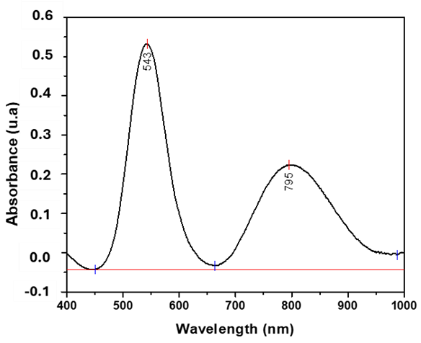 | 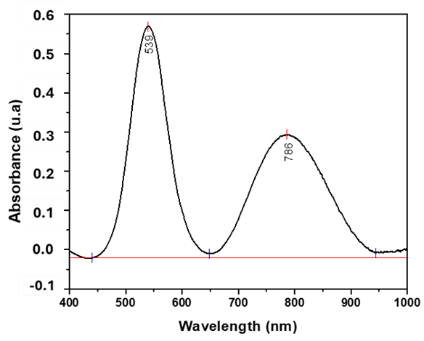 |
| G. | H. | I. |
| 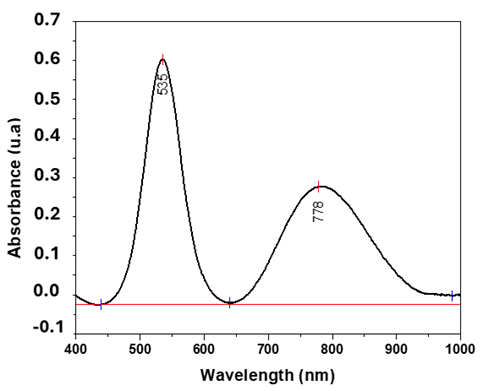 | 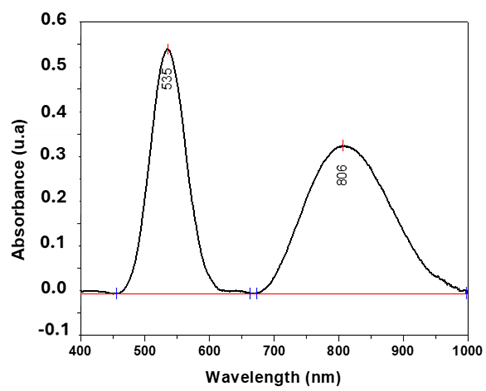 | 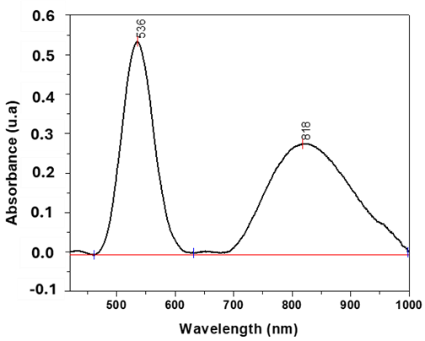 |
| J. | K. | L. |
| 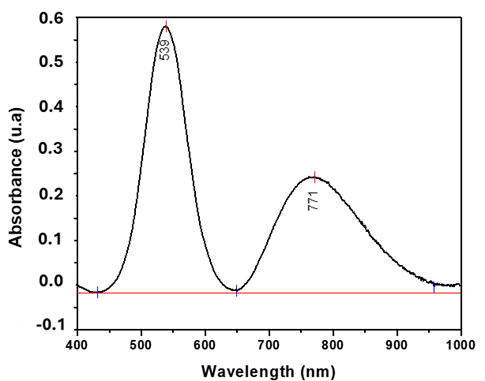 | 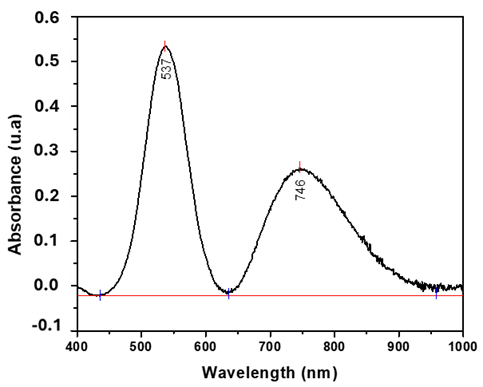 | 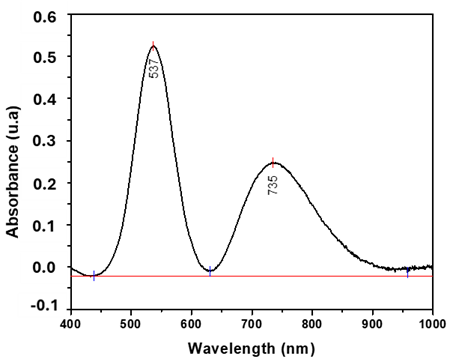 |
| M. | N. | O. |
| 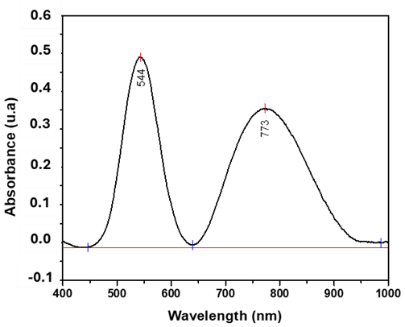 | 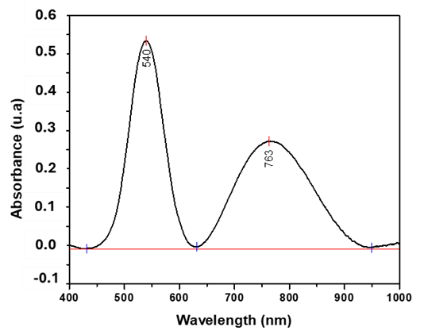 | 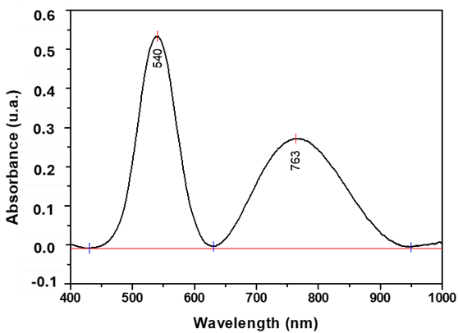 |
| P. | Q. | R. |
| 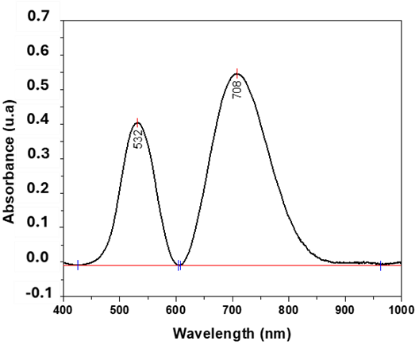 | 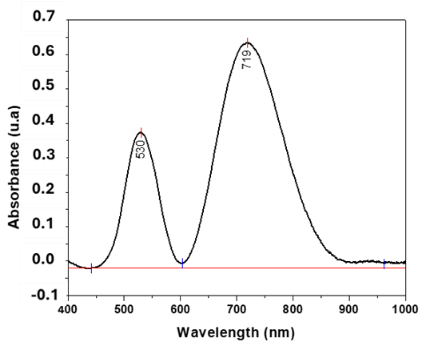 | 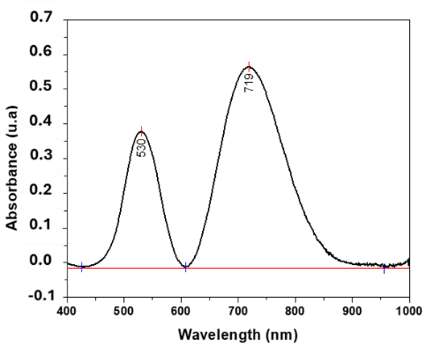 |

Supplement: S1 Fig — (DOCX) [file pone.0313485.s002.docx]
